# Pruritus in pediatric patients with atopic dermatitis: a multidisciplinary approach - summary document from an Italian expert group

**DOI:** 10.1186/s13052-020-0777-9

**Published:** 2020-01-30

**Authors:** May El Hachem, Giuseppe Di Mauro, Roberta Rotunno, Simona Giancristoforo, Cristiana De Ranieri, Carla Maria Carlevaris, Maria Carmen Verga, Iride Dello Iacono

**Affiliations:** 1 Dermatology Unit, Bambino Gesù Children’s Hospital, IRCCS, Piazza di Sant’Onofrio, 4, 00165 Rome, Italy; 2 Primary Care Pediatrics, ASL Caserta, Caserta, Italy; 3 Unit of Clinical Psychology, Ospedale Pediatrico Bambino Gesù (IRCCS), Rome, Italy; 4 Bambino Gesù Children’s Hospital, IRCCS, Rome, Italy; 5 Primary Care Pediatrics, ASL Salerno, Vietri sul Mare, Italy; 6 Unit of Pediatrics, Fatebenefratelli Hospital, Benevento, Italy

**Keywords:** Atopic dermatitis, Pruritus, Multidisciplinary approach, Therapeutic patient education

## Abstract

Given the inadequate overall awareness of the main disease features and treatment modalities of pruritus in pediatric patients with atopic dermatitis, a multidisciplinary Italian expert group met with the major aim of increasing knowledge of the condition for improved diagnosis and better management among specialists involved in disease management. Herein, the overall features of the condition are reviewed, along with its etiopathogenesis and symptoms. Likewise, management options are summarized, emphasizing the need for a multidisciplinary approach, minimally composed of a management team that includes a pediatrician, dermatologist, psychologist, play assistant, and dedicated nurse. In addition to more traditional therapies such as emollients as highlighted by European guidelines, therapeutic patient education in a group or individually is highly encouraged as it helps patients and their parents to better understand the disease and provide practical guidance for dressing and bandaging. It can also aid in outlining coping strategies for itching and sleep disturbance. The utility of distraction techniques should also be stressed as such educational interventions involving the child and their parents can substantially improve the overall quality of life. All approaches should be tailored according to patient age and clinical features and requires individualized strategy to ensure good adherence by both children and their parents. Thus, a holistic approach embracing systemic, topical and psychological interventions is advocated in order to provide patients and their caregivers the best possible care.

## Introduction

Atopic dermatitis (AD) is a chronic multifactorial disorder that requires multidisciplinary management [1, 2]. The recent literature confirms that the treatment of itching is a challenge. Unfortunately, opinions and approaches are discordant among physicians from the same or different specialties. This can cause confusion for patients and families with implications for decreased adherence to treatment and worsening of disease complications such as lichenification, infection, and sleep disturbance, which negatively affects the overall quality of life (QoL). The therapeutic approach should be adapted to patient age and clinical features and requires a patient-tailored strategy to ensure good compliance by both children and their parents. Considering this aim, an Italian expert group has developed this document as a tool to different specialists involved in disease management (pediatrician, dermatologist, allergologist, psychologist, psychotherapist, play assistant, and nurses) to plan adequate and tailored treatment for each AD patient and itching. Herein, we provide a concise summary document that is relevant for practicing clinicians.

### Clinical features and differential diagnosis of AD

AD is most common during childhood [2]. The diagnosis of AD is generally easy and based on clinical features that vary upon patient age and disease severity (Table 1). In addition, other signs and symptoms may be associated and are helpful to confirm the diagnosis in some patients (Table 2). Differential diagnosis is always necessary and arises in a list of diseases from variable severity requiring different management approaches (Table 3).

**Table 1:** Characteristic features of atopic dermatitis by age. Modified from Ref. [1]

AD	Infant	Childhood	Adolescent or Adult
Lesions	Exudative erythematous weepy papules and plaques	Weepy erythematous papules and plaques intermixed with lichenified plaques, particularly in flexural areas	Erythematous papules and plaques with xerotic scale and crust Lichenified plaques in flexural areas
Distribution	Scalp, face (without perioral and periorbital involvement) trunk, extensor surfaces	Flexural surfaces, including antecubital and popliteal fossa, wrist, and neck	Hands, flexural surfaces, upper trunk

**Table 2:** Associated features of atopic dermatitis. Modified from Ref. [1]

Pityriasis alba: Hypopigmented patches on face, upper trunk, upper extremities	
Keratosis pilaris: Follicular hyperkeratosis of outer arms, lateral cheeks, buttocks, thighs	
Dennie-Morgan fold (atopic pleat): Extra line on lower eyelid	
Allergic shiners: Violaceous to gray color of infraorbital area	
Hyperlinear palms: Increased and exaggerated skin markings on palms	
Ichthyosis vulgaris: Scaling of extensor extremities, fish-scale appearance of extensor leg	
Hertoghe sign: Loss of lateral eyebrows	
White dermatographism: Blanching of skin after stroking	
Circumoral pallor: Pallor of perioral area	
Nummular dermatitis: Sharply circumscribed thick coin-shaped scaly plaques

**Table 3:** Atopic dermatitis and differential diagnosis. Modified from Ref. [1]

Disease	Age	Morphology	Distribution	Symptoms
AD	Childhood	Erythematous eczematous weepy plaque with fine dry scale	Face, scalp, Extensor surfaces in infants, flexural surfaces in children, trunk, nails	Severe pruritus
Seborrheic dermatitis	Any age	Salmon-pink fine papules coalescing into poorly defined plaques with variable greasy scaling.	Face, scalp, Retroauricular folds, neck, axillae, inguinal folds	Absent to mild-moderate pruritus
Psoriasis	Any age	Well-demarcated, pink plaques with adherent silvery scale, confluent bright red plaque with sharply defined borders and relative lack of scale.	Extensor surface of joints (elbows, knees) and extremities, Retroauricular folds, axillae, umbilicus, diaper area, inguinal folds, genitalia, gluteal cleft, nails	Mild-moderate pruritus
Allergic contact dermatitis	Any age, incidence increases with age	Geometric erythematous eczematous weepy plaques	Based on exposure	Severe pruritus
Scabies	Any age	Poorly defined erythematous papules, nodules, burrows, pustules, and vesicles	Wrists, interdigital spaces, axillae, umbilicus, nipples, diaper area,	Severe nocturnal pruritus
Mycosis fungoides	Adulthood, Hypopigmented MF More common in children	erythematous patches, papules, or plaques with subtle scale; polycyclic or annular, hypopigmented patches, often with fine scaling	Buttock, lower trunk, thighs, breasts, and groin	Pruritus
Dermatomyositis	Childhood and middle age	Violaceous scaly papules, Periorbital violaceous edema	Small joints of hands and elbows, face	Myositis
Immunodeficiency and metabolic disorders	Infancy	Eczema or eczema-like eruption, erythroderma	Spread, resistant to treatment	Other cutaneous and extracutaenous manifestations Blood testing and genetic investigations upon diagnostic hypothesis

### The etiopathogenesis of itching in AD

**Fig. 1:**
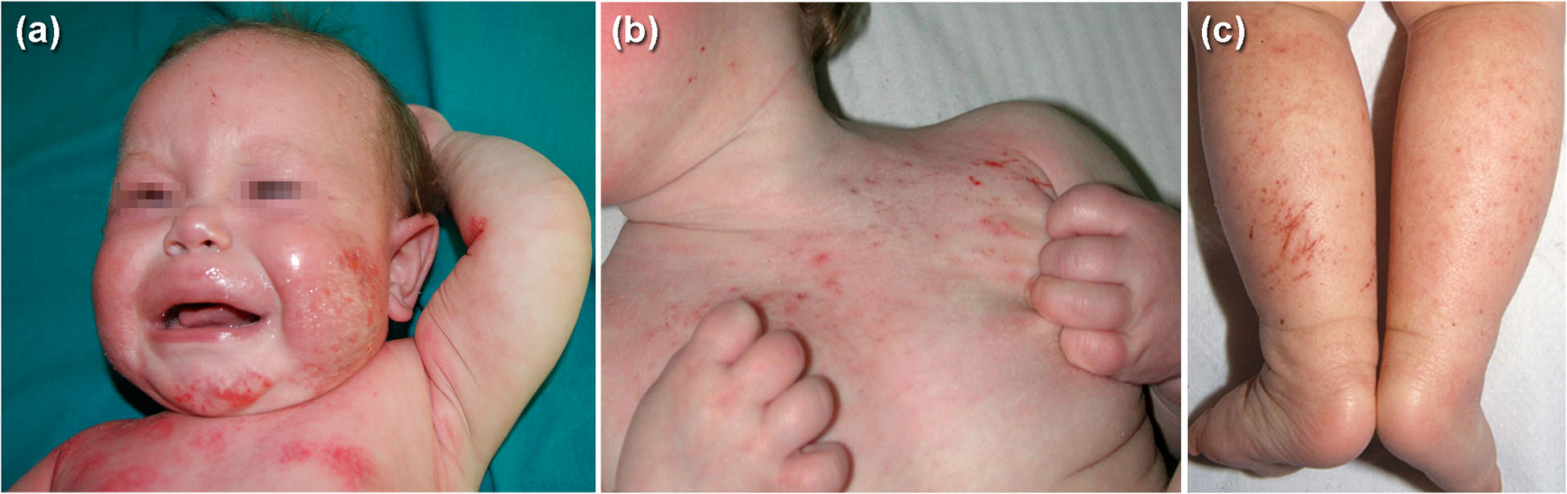
**a** An infant aged 6 months affected by atopic dermatitis, exudative lesions on the cheeks and chest, and crying because of severe itching and pain. **b** Small babies even 2 and 3 months of age may scratch causing erosions. **c** scratching lesions on the legs

Pruritus is the major and disrupting symptom of AD and is due to a complex interaction comprising skin barrier deficiency, immunological dysfunction, and central and peripheral neural mechanisms. Itching promotes a negative cycle described as “the itch that rashes” [2, 3]. Scratching causes further damage to the skin barrier, stimulating skin inflammation and inducing factors called pruritogens. (Fig. 1) This mechanism leads to the development, progression, chronicity of AD, and complications [3, 4]. The main etiopathogenetic factors are summarized below as follows:

- Skin barrier disruption. AD is characterized by systematic epidermal barrier dysfunction; due to, at first, tight junction aberration with reduced expression of some claudins and zonulins. In addition, several mutations in the filaggrin gene have been identified. This filament is essential for controlling transepidermal water loss (TEWL) and maintaining stratum corneum organization and hydration [3–5]. Itch intensity in AD is associated with increased TEWL. Filaggrin deficiency also leads to an increase in cutaneous pH, which enhances the function of kallikreins, or serine proteases known as pruritogens that are upregulated in AD [3, 6]. Moreover, epidermal barrier dysfunction allows for entry of irritants and pruritogens [6, 7].
- Immunological disorder. Skin barrier dysfunction and inflammation lead to an aberrant type 2 immune response, with increased IgE production, eosinophilia, mast cell activation, and overexpression of Th2 cytokines (IL-4, IL-5, IL-13). This cascade promotes production of epithelial-derived cytokines, namely, thymic stromal lymphopoietin (TSLP). TSLP influences innate lymphoid cells (ILCs) and increases the production of Th2 chemokines [4, 5, 8].
- Hyperinnervation of skin and central sensitization of itch. An increase in nerve fiber density in the epidermis reported in AD, which is partly explained by the increase in nerve growth factor observed in the plasma of AD patients. Moreover, some studies have implicated involvement of central nervous system structures and astrocytes in sensitization of itch. Other studies have revealed a central neural circuit that is critical for signal processing of itch [9, 10].
- Itch mediators/pruritogens**.** The sensation of itching is mediated by cytokines, neuropeptides, and endogenous secreted factors. These itch-inducing factors act on sensory neurons to drive itch sensation. Histamine is one of the earliest identified pruritogens; among four receptors, H1R and H4R are potential mediators of pruritus. Others endogenous and exogenous factors produced from inflammation and xerosis result in the induction of non-histaminergic itch (i.e. protease astryptase, dust mites, *Staphylococcus aureus*, or substance P, TSLP, Notch proteins) [5–7, 11]. Recently, a role of IL-31 produced by Th2 cells has been recognized in inducing itch [12, 13].

### Symptoms and complications

#### Symptoms

Pruritus can occur in different clinical forms depending on the age of the child, chronicity of symptoms, trigger factors, and psychological attitude. In AD, itching is the main, constantly present symptom, and is of variable intensity, associated with pain and/or burning, and generally worsens at night causing sleep disturbance.

Correct classification of symptoms and emotional consequences, due to a vicious circle, is essential to plan adequate management [14]. In the literature, there are 62 separate severity scales and 28 quality of life tools (QoL) used in clinical research; however, only 3 have been properly validated [15]. The most commonly used scales are the SCORAD index, the Eczema area and the Severity Index (EASI), the global assessment of the investigator (IGA), and the SASSAD (Six Area, Six Sign Atopic Dermatitis) [16].

The tools used for assessment of the intensity of itching are the Visual Analogue Scale (VAS), Numerical Scale, Verbal Scale, and Itching Severity Scale (ISS). However, there is no gold standard instrument for accurate assessment of the intensity and severity of pruritus and it is widely recommended that at least two should be used for reliable judgment [17].

#### Complications

**Fig. 2:**
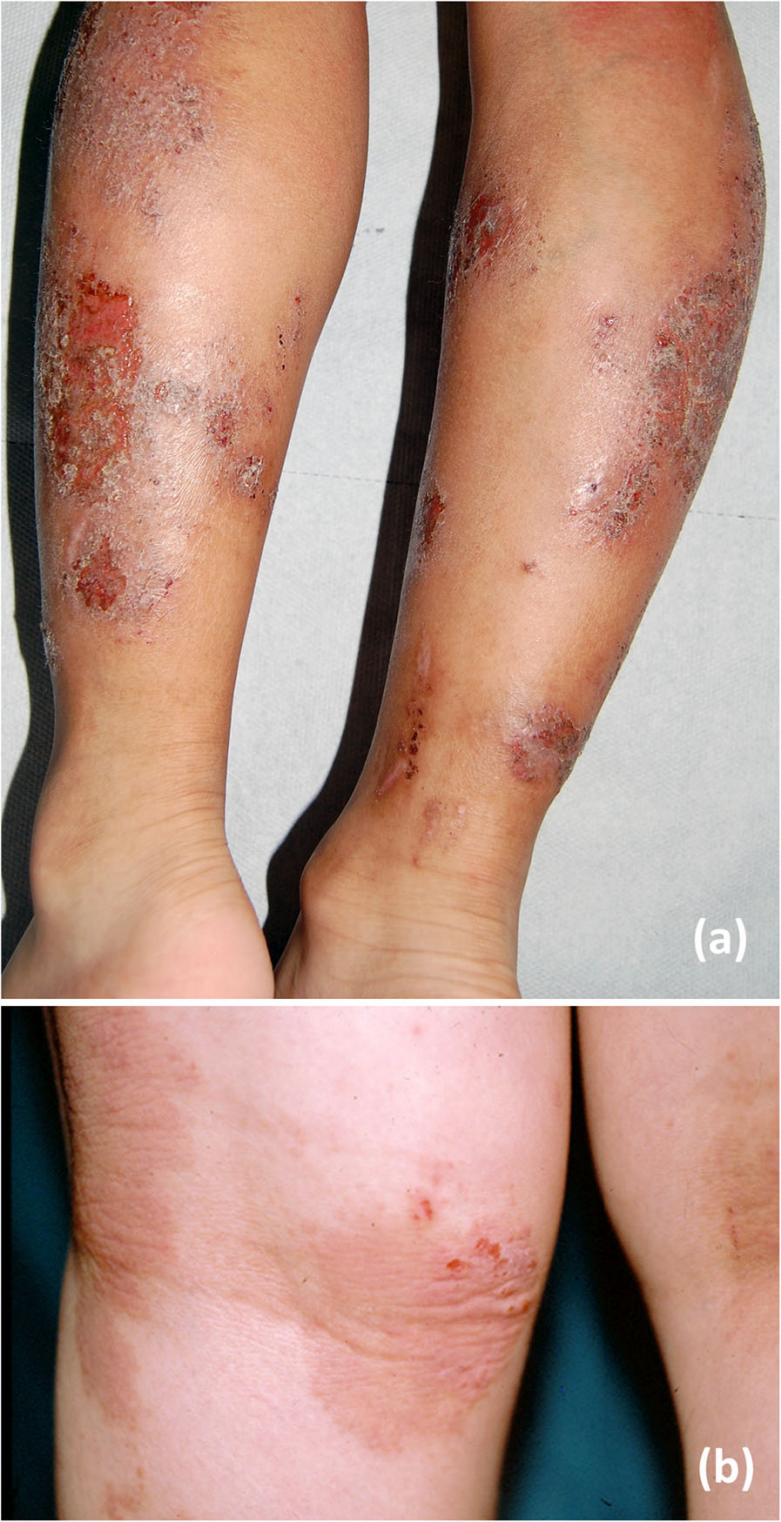
**a** Erosions on the legs of a 9 years aged boy with AD, due to severe scratching, in this case there is a high risk of infection. **b** Chronic scratching in a girl with AD causing lichenification on the flexural regions of the left knee

In patients with DA, constant pruritus can lead to chronicity with the appearance of nummular eczema, lichenification and/or nodular prurigo. Bacterial and viral superinfections are a frequent complication due to altered skin barrier and itching. In addition, scratching may induce autoinoculation, particularly in the presence of molluscum contagiosum and impetigo [18].(Fig. 2).

The persistence of pruritus, sleep disturbance, and worsening of skin conditions have a negative impact on psychological development of the child and on the emotional status of both the child and his/her parents, affecting daily activities, social relationships, and quality of life. In some cases, care to the AD patient may generate a conflict between the parents and the non-affected children, who may feel neglected.

In some situations, targeted psychological intervention may be necessary. It is appropriate to suggest this in the presence of: (i) strongly involved parents (disproportion between the problem and its consequences), (ii) excessive influence of the disease on daily life and interpersonal relationships, (iii) parent/child relationships characterized by mutual dependence, and (iv) psychological issues in a family member or in the functioning of the family nucleus.

### Therapeutic patient education

The WHO defines therapeutic patient education (TPE) as a continuous process to train and guide the patient and his/her family to acquire the competencies needed to manage his/her chronic disease. TPE is mandatory for adequate management of AD and itching for several reasons:

- AD is a multidisciplinary disease with discordant opinions between specialists
- clinical features are polymorphic in the same day in the same patient
- early age of disease onset
- lack of specific treatment against pruritus
- sleep disturbance involving the family
- scarce compliance due to chronicity, time and costs for dressing
- esthetic damage
- psychosocial impact

TPE should be performed within the multidisciplinary approach required for AD. The specifically trained team should be minimally composed of a pediatrician, dermatologist, and dedicated nurse. In some cases, a psychologist is also required. All specialists should use the same language and provide the patient/family with a specific and tailored educational program. When required, other professional categories (e.g. allergologist) should be informed and take part in the TPE.

TPE can be performed within a group or individually and comprises:

- a theoretical aspect explaining the disease, its evolution and stressing its benign prognosis
- providing specific educational leaflets to the parents and children with adequate language
- a practical demonstration of dressing, patient massage, bandaging etc.
- strategies and tools for coping with itching and sleep disturbance (such as distraction techniques and their illustration)
- continuous evaluation of the patient and family perception
- psycho-diagnostic testing and psychotherapeutic intervention, when required.

A well-integrated and coordinated TPE program ensure adherence to treatment and better control of pruritus and sleep disturbance, thus reducing overall costs and improving QoL [19, 20].

#### Medical treatment

Antipruritic therapy is multidimensional aimed at minimizing symptoms and contributing factors such as dry skin, inflammation, and related scratch lesions. Therefore, several measures can be recommended [16, 18–20]. Treatment of AD improves itching. Evaluation of the efficacy of different therapies on itching is difficult due to multiple components influencing this symptom, including psychological attitude.

Immunotherapy to inhalants is indicated in addition to other topical and/or systemic treatment in order to improve itching and please add the reference [21].

#### Topical therapy

Topical therapy is mandatory to repair skin barrier and reduce itching and skin susceptibility to irritants. Hydration is always essential, even during remission. Several topical products are available, and the selected formulation should be cosmetically acceptable to the patient, and possibly inexpensive, in order to encourage good compliance. The specific choice of treatment depends on both individual and clinical features [16, 22–24].

##### Topical skin care

For all topical skin care, it should be highlighted that specifically formulated products should be used, which are free of preservatives and perfumes [25].

- Cleansers. Oil, non-soap, or mild soap cleansers, without perfumes. Sodium hypochlorite (bleach) baths as antiseptic is an additional option [23, 24].
- Hydration. Emollients and moisturizers are a mainstay of pruritus management. As undressing could deteriorate itching, emollients should be applied on xerotic/lichenified skin not covered by clothes twice daily, and at least once daily to the entire surface of the skin, and more when undressing children for other reasons. They should be regularly applied, even during remission to prevent itching, flares, and complications [22, 23, 26, 27]. Emollients “plus” are non-medicated products containing active ingredients [28, 29].

##### Anti-inflammatory therapy

- Corticosteroids: topical corticosteroids (TCS) are still considered the mainstay of pharmacological treatment and first choice agents for inflammation and itching [18–20, 30, 31].
- Calcineurin inhibitors: tacrolimus ointment (0.03 and 0.1%) and pimecrolimus cream (1%) are immunomodulators used for moderate/severe and mild forms, respectively. It is important to take into account the age limit and possible contraindications (immunosuppression, exudative and infected lesions) [18–20, 32, 33].

##### Antimicrobial therapy

- Antimicrobials and antiseptics are necessary to treat infected lesions. Fusidic acid, retapamulin, and mupirocin are the most appropriate antibiotics [34, 35].

##### Upcoming topical therapies

- Topical phosphodiesterase 4 inhibitor, crisaborole, is an effective treatment of mild-to-moderate AD in patients from 2 years of age and is still experimental in Italy [36].
- Topical Janus kinase (JAK) inhibitors are being studied in clinical trials [37].

##### Wet dressing, or wet-wrap therapy

- The application of a topical medication followed by bandaging or wet dressing (a double layer of gauze or tubular dressings, the first layer moistened and the second layer dry) is indicated in exudative, infected or lichenified lesions, and protects against scratching and environmental irritants. It is also useful to promote absorption of topical products. Thus, it is important to take into account possible side effects, especially with concomitant corticosteroids [38, 39].

#### Phototherapy

UV therapy can be used in AD to relief pruritus. Narrowband UV-B and UV-A has been demonstrated to be the most preferable artificial radiation [40, 41].

#### Systemic therapy

Systemic therapies with confirmed effects against pruritus recommended by the guidelines are:

- Antihistamines. Short-term and intermittent courses of sedating (first-generation) antihistamines can be used in children if itch strongly affects quality of sleep [42–44].
- Systemic corticosteroids. Short courses of therapy may be indicated in special situations [45, 46].
- Systemic antimicrobial therapy. Antibiotics should be reserved exclusively for clear signs and symptoms of bacterial infection, and not simple bacterial colonization [45, 47].
- Oral immunomodulatory therapies. These are to be used for chronic itching recalcitrant to topical therapy [45, 48–51].
- Target therapy. Biologic agents against pathogenic cytokines and their receptors are a relatively new group of therapeutics [45]. Dupilumab (anti IL-4 and 13) is recommended as a disease-modifying drug for moderate-to-severe AD in Europe [52]. Treatments with ustekinumab (anti IL-12 and IL-23), tralokinumab (anti IL-13), or nemolizumab (anti-IL-31) are still experimental [53–56]. Oral drugs such as TSLP antagonists and JAK inhibitors are under development [57, 58]. Their use should be restricted to very selected cases and used in specialized centers.

##### Therapeutic patient education follow-up and psychological support

Despite the increasing prevalence of childhood AD, only a few studies have explored a specific educational approach in the last years. Recent national and international AD guidelines confirm that effective and standardized educational programs should be considered part of AD management and routine care. These treatment strategies are focused on patients and their families. Interventions must be delivered by a multidisciplinary team with an individual face-to-face or group education. The need for education and support will vary according to patient age, socio-cultural behavior, and severity of AD. The educational approach should be preferably focused on families of children below 6 years of age because of the significant and high impact on disease and allow good management following disease onset. It is clear that the motivation to treat is high if the parents receive more (and correct) knowledge about the disease. Parents who play a key role in their children’s treatment can considerably reduce the severity and impact of AD. This suggests that in addition to pharmacological treatment, providing educational support to parents is an important factor in achieving a positive long-term outcome. Less benefit for children more than 6 years with moderate or severe eczema has been reported because they become with time less motivated to treat themselves [59], and for this reason the educational program should start as soon as possible. The treatment of AD should ideally take into consideration both the patient’s physical and emotional aspects and the impact of the disease in personal and family contexts. Attention to the emotional area allows: (i) promotion of a psychological elaboration that integrates information and emotions, facilitating memorization and consolidation of new information; (ii) creation of a relational context in a dedicated area for listening to the patient and his/her parents (knowing what he/she knows, how the patient lives with the illness, how much it influences them and their family); (iii) supporting the resources and skills of the patient and parents. An early start of the training process is important, before any feelings of ineffectiveness and mistrust and/or relationship difficulties and conflicts are established. For management of AD and pruritus, we think that education should be based on an approach that addresses the needs of the child and parents at a level they can comprehend. Educators are dermatologists, pediatricians, nurses and psychologists. The educators’ role with the intervention group is to train, support, and motivate parents and children in self-management. One of the most important interventions in the management of AD is spending time listening to patients’ concerns, explaining its causes, and showing how to apply topical therapies on the basis of clinical features of the lesions and patient age. The application of topical therapies is an essential part of the successful management of pruritus and atopic eczema. Longer consultations and forming a good caregiver-patient relationship are the strongest predictors of adherence to skin-care treatment. Performing this role requires well-trained educators [60].

##### Adjuvant therapies: distraction techniques

Treatment of children with AD and pruritus and adequate management of the daytime and nighttime problems due to itching have a strong impact on the entire family context, making it necessary to focus on emotional and relational aspects. In order to facilitate the management of pediatric patients during application of dressings and to provide tools to deal with frequent itching, the use of specific distraction techniques is crucial. These non-pharmacological procedures with a psychological approach have been described in the literature [61–63], and are especially useful in pain management and during unpleasant procedures and or sensations. These are cognitive-behavioral techniques, aimed at shifting attention from pruritus and medication towards activities that intensely absorb the patient: the techniques imply active sensory perception, and not just a passive strategy, that can be entertaining the child. The techniques are based on two principles: (i) children have mental boundaries between fantasy and reality that are more fluid and permeable than in adults; (ii) and it is difficult, if not impossible, for a human to focus on more than one or two sensory stimuli at once. A detailed description of distraction techniques by age group along with specific examples is provided as Additional file 1.

The choice of distraction techniques (involvement, breathing, relaxation, hypnotic procedures such as visualization and desensitization) depends on several factors: age, personality, individual likes and dislikes, motivation, and emotions of reference figures. In addition, even the time of day is important since, for example, at night a massage would be favored over watching a cartoon. The application of these tools allows defusing the phenomenon of anticipatory anxiety and increased compliance to treatment. These techniques are useful not only for children, who are the main recipients, but also for parents and siblings. It is therefore important to educate the entire family on their use, and to adapt them in specific situations. Distraction techniques are also helpful to parents to feel more relaxed, they improve the interaction with their child, transmitting greater security and managing the disease with less anxiety. The utility of learning techniques has also been documented in the parents of very young children [59–62].

## Conclusions

The focus of the present summary document was to increase awareness of pruritus in pediatric patients with AD. In addition, the authors tried to provide a useful tool for all specialists who managed patient with this disease and pruritus. Emollients, as reported by the literature are considered the backbone in restoring the skin barrier, reducing itching and relapses. The psychological support should be contemplated as one of the several therapeutic options available or on trial. Finally, it is emphasized that a multidisciplinary approach is always necessary to guarantee an adequate management of patients with AD. Moreover, educational interventions can greatly improve reducing symptoms, improving the adherence to treatment and the overall quality of life for both young sufferers and their parents.

## Supplementary information

**Additional file 1:.** Descrition of distraction techniques by age group.

## Data Availability

Data sharing not applicable to this article as no datasets were generated or analyzed during the current study.

## Abbreviations

AD: Atopic Dermatitis
EASI: Eczema Area and the Severity Index
IGA: Investigator Global Assessment
ILCs: Innate Lymphoid Cells
ISS: Itching Severity Scale
QoL: Overall Quality of Life
SASSAD: Six Area Six Sign Atopic Dermatitis
SCORAD: SCORing Atopic Dermatitis
TCS: Topical Corticosteroids
TEWL: Transepidermal Water Loss
TPE: Therapeutic Patient Education
TSLP: Thymic Stromal Lymphopoietin
VAS: Visual Analogue Scale
