# Supplementary material for: Pruritus in pediatric patients with atopic dermatitis: a multidisciplinary approach - summary document from an Italian expert group
Source: Ital J Pediatr. 2020 Jan 30;46:11. doi: 10.1186/s13052-020-0777-9 (PMC6993480; doi:10.1186/s13052-020-0777-9)
Supplement: Supplementary file 1 — Additional file 1:. Descrition of distraction techniques by age group. [file 13052_2020_777_MOESM1_ESM.docx]

**DISTRACTION TECHNIQUES**

To help with the management and control of unpleasant sensations related to dressings and pruritus in children with atopic dermatitis, several non-pharmacological techniques are available that can be helpful.

These techniques have shown effectiveness in the management of acute and chronic pain, and should not be considered as passive strategies oriented to entertain or deceive the child, but rather as active strategies aimed at focusing attention on an alternative stimulus: *the child must be intensely absorbed in an activity, and for children it can be considered as a high energy game.*

These techniques are based on two principles that allow one to use the remarkable imaginative skills of children:

- boundaries between fantasy and reality are much more fluid and permeable in children than in adults, amplifying their ability to use their fantasy
- it is difficult, if not impossible, for a human being to focus attention on more than one or two sensory stimuli at once.

These techniques imply the involvement of **parents** and, as soon as possible, as well as siblings, so that the dressing becomes a moment of sharing that stimulates family relationships.

They can be used during dressing or when the itching becomes unbearable, if the child is already familiar with dressing that they can applied more easily and automatically use the techniques when needed.

**CHOOSING THE TECHNIQUE**

The choice will consider the **age of the child, his tastes, personality and the time of day in which intervention is needed.**

Breathing and relaxation are more suitable for children with concentration and self-control skills. Distraction is more suitable for more lively and extroverted children. As the child becomes more familiar with breathing and relaxation, the techniques can be skipped or briefly summoned as needed

The following list summarizes the adaptability of the techniques to different age groups. Individual differences must be considered. Each technique can be used in a more elementary or more complex way.

The main techniques are breathing, relaxation, distraction, visualization, guided imagery, and simple techniques using hypnoanalgesia such as the 'red cloud', the 'magic glove' and the 'switch'. (explained in the following pages)

| 0-2 years | Physical contact: touch, caress, rocking.  Listen to music, show toys above the cradle. Playful approach, soap bubbles.  Sensory saturation*: saturate the senses of the newborn by drawing attention to positive stimuli (tactile, auditory, visual or gustatory): the newborn is placed with the knees flexed but without restriction. The infant it is observed very carefully, speaking gently, massaging the face and body and glucose is given.* |
| --- | --- |
| 2-4 years | Breathing techniques (with soap bubbles), distraction, involvement, simple visualization, imagination, magic glove |
| 4-6 years | Breathing techniques (with soap bubbles), distraction, involvement, visualization, desensitization (magic glove, switch). |
| From 6 years | Breathing, relaxation, distraction, involvement, visualization, desensitization |

**TECHNIQUES**

**BREATHING**

**From the age of 3**, you can ask the child to breathe slowly and deeply: inhale deeply and feel their muscles relax, and then exhale naturally inflating the lungs, feeling the air coming in and out.

Example:

*“Take a deep breath until your stomach lifts up a little, hold your breath and then exhale. With exhalation, you feel relaxed. To help you concentrate, imagine yourself counting: in, two, three; out, two, three.*”

**For young children (< 3 years)** this can be taught together with distraction techniques.

*Breathe deeply by blowing soap bubbles*: soap bubbles combine breathing (the child must activate the exhalation to produce them) and distraction (the child becomes easily enchanted by the number, shapes, colors, and movement)

*Red Cloud:* You can encourage the child to throw out fear and pain with a red cloud. It combines breathing and visualization, inviting the child to concentrate every unpleasant feeling in a cloud that becomes increasingly red and that is blown away with all possible energy. One can imagine that it turns into something different, it can shrink or enlarge, and the color can become increasingly lighter as it moves away.

**RELAXATION**

**From 4-5 years** relaxation can be taught together with breathing: the child is invited to feel their muscles and to release them to become like a pudding, soft and relaxed. It proceeds progressively, starting from the muscles of the neck, then passing to the shoulders, chest, belly, and up to the arms and legs.

Relaxation is proposed to amplify the sensations connected to the parts of the body where one’s attention is focused and highlights the differences between tension and the possibility of letting it go. It can be usefully combined with some strategies of autogenic training: focus and gradually associate feelings of heaviness, warmth, or color to the different areas on which attention is concentrated.

Sometimes, for example, when the child is stressed due to changes in the body, this technique is not indicated. It is better to focus attention not on the body, but on the outside using a reassuring scene and then use a distraction technique.

Example:

*“Get as comfortable as possible, move until you feel comfortable. Close your eyes when you're ready. Take a deep breath with your nose and throw it out completely, breath in again and breath out slowly.*

*Focus on the hand, clench your fist and then release it. Feel the difference.*

*Do the same with the muscles of the arm: point the elbow, push hard and relax the arm. Raise your eyebrows as hard as you can and then let them go. Wrinkles your nose and then relax and let go. Smile and then release. Lower your chin to your chest, keep your muscles tight and then and let go. Bring your shoulders back and let go. Raise your legs and toes to the ceiling and let go.*”

**DISTRACTION**

It is simple, intuitive, and immediate to implement without age limits: it involves the use of the child’s preferred objects (three-dimensional books, puppets, videos, games) that captures their attention away from the fear and anxiety of pain, and mentally engages them in activities that they like.

It can be active, playing with something, or passive, watching a cartoon.

Examples:

*Name and describe objects in the room or images from a magazine.*

*Tell a story without worrying about the ending, but concentrating in alternating the narration in a playful way. Some ideas for imagining stories together: identify a fictional or well-known character who gets into different situations from time to time; overturn a story that is already known, change the age of the protagonist, the place, or the conclusion (what would happen if Snow White does not eat the apple?).*

*Word games: i) think about an object, an animal or a person, that the other person guesses but can only ask questions that can be answered with yes or no; ii) word chain, where each person says a word that is associated with the previous one; iii) find a rhyme with the word chosen by the other person and create rhymes; iv) a word is chosen and for each letter each person finds an adjective appropriate for that word (sun: shiny, unique, natural).*

*Mime game: representing a profession, title of a film, name of a character, without using words but through body movements.*

**INVOLVEMENT**

Every child is different, and some need to have the situation under control. A form of distraction is involvement: it engages the child during the medication to divert attention through direct control of physical sensations.

Children who actively participate in these procedures are distracted from the discomfort and concentrate on the action to be taken.

Examples:

*While the mother applies the dressing to the child, the child applies it to the doll. You can give the baby some cream to spread and the gauze to apply to the doll. Brothers and sisters can be involved with their dolls. The important thing is that the dolls are all made of plastic and easily washable.*

*From 5 years onward, during daily dressing changes, the child can be involved in the process to learn how to do it to make it part of their daily habits. Give the child a little cream in his hands and let him try the different sensations (from cold to warm, slippery skin, absorption, smoother skin after treatment).*

**VISUALIZATION**

One can use visualization, or guided imagery, from 5 years of age onwards. The focus is on breathing and relaxing the body and moving to deeper levels of consciousness, where the conscious mind can access a greater number of images.

After relaxation, the child is guided to imagine a pleasant situation and/or place in which they would like to be and to imagine themselves in that place. It is important to create a multisensory experience in which the child uses all 5 senses by asking if they can see details such as colors, if they smell perfumes or hear sounds or noises, if they can touch something or taste flavors.

Example:

*“Get comfortable ... take a breath and breath out slowly. Relax your body in all its parts ... imagine being away from this room, in a beautiful place. You're on a path at the beginning of a forest, there are tall trees and low plants, a light wind blows, the sun’s twilight enters the branches ... see the light, feel the vibration on the skin ... Continue along the path, calmly.*

*Look at the colors, green, orange ... smell the scents ... listen to the sounds of the wind and the leaves and the birds ... Walk more and more into the forest, at some point the path opens into a small clearing and a little hill with the entry of a cave is in front of you. Enter the cave. There is a small rock, you sit down and rest. While you are there a person arrives. A wise and welcoming figure, he welcomes you, tells you a few words, hear the tone he uses. He gives you a gift and puts it in your hands ... thank him and tell him that you will keep this gift with you. He tells you that you can come back every you want. Go out and back to the path, walk back towards the exit of the woods. Return: now when you're ready, go back to the room, 3-2-1 ... feel the chair or the bed ... open your eyes ... whenever you want you can come back to the place that makes you feel good”*

*The child can be invited to go to their favorite place, real or imaginary, or they can use different guided imaginations: invite them to fly on a magic carpet and repeat what they see, explore a secret garden, open a door and see what behind, take a trip on an unknown planet, swim close to the seabed, enter a magical shop and describe what you find, an adventure with your hero ...*

*Or imagine doing one of their favorite activities, like playing soccer, swimming, making a cake, etc.*

*Or to remember the funniest film they’ve ever seen, projecting it in their mind.*

**DESENSITIZATION**

This is a more complex process. The child must be prepared gradually: first, breathing and relaxation, then driving ones concentrating to the painful area.

The child, through his concentration, manages to lower the intensity of itching in a specific area of the body.

Examples:

*The most used techniques with children are:*

*- objectification and identification: visualizing a large circle in front of oneself, representing one's discomfort or negative emotion, imagining its color and distance gradually changing.*

*- use of metaphors: what is the itching like? A bird that pecks? Fire? Can you make the bird fly away?*

**Magic Glove**

It simulates wearing an invisible glove (or magic sock at the foot, magic kneepad, ...). The child chooses the color and wears it with very realistic movements: thus, they will perceive a sort of numbness in their hand, lowering their sensitivity. Once finished, it is important to remove the magic glove to restore sensitivity. Carefully take off the glove and store it for the next time.

**Switch**

Over 9 years of age

After relaxation together, the child is invited to visualize a switch in his mind that can relieve the discomfort of the itching.

Once the switch has been identified, the child is told to look at its colors and shape and try to lower it step by step. As the level of the switch decreases, so does the itching.

Example:

*“Focus and enter the control room. There are all the switches that control all the parts of your body. Find the switch in your head that commands the part of the body that annoys you. Give me a nod when you have found it. Note the color and the shape of the switch and lower it. Inch by inch, at each level you notice the itching decreases. Keep lowering it and you will feel much better”.*
